# The role of guided growth as it relates to limb lengthening

**DOI:** 10.1007/s11832-016-0779-8

**Published:** 2016-12-02

**Authors:** Peter M. Stevens

**Affiliations:** Department of Orthopaedic Surgery, University of Utah School of Medicine, Salt Lake City, UT USA

**Keywords:** Epiphysiodesis, Guided growth, Limb lengthening, Anisomelia

## Abstract

For decades, the classic indication for limb lengthening has been reserved for anisomelia that was expected to reach or exceed 5 cm at maturity. Epiphysiodesis was reserved for discrepancies in the 2–5 cm range. With the increasing sophistication of fixators, including rail, hexapod, and hybrid, complex deformities may be corrected simultaneously while moderate to extreme lengthening is achieved. More recently, iterations of telescoping intramedullary rods have further strengthened our armamentarium. Meanwhile, permanent epiphysiodesis techniques, both open and percutaneous, have yielded to more versatile and reversible tethering of one (angle) or both (length) sides of a physis. While the techniques of guided growth and callotasis seem to be diametrically opposed, they may be used in a tandem or complementary fashion, for the benefit of the patient. If treatment is undertaken during skeletal growth, one must be aware that issues remain regarding the accurate assessment of skeletal maturity and prediction of the ultimate outcome. Therefore, there is potential for over- or undercorrection. Reversible and serial guided growth now enable the surgeon to commence intervention at a comparatively young age, for the purpose of optimizing limb alignment and reducing the ultimate discrepancy. Frame application may be delayed or, in some cases, avoided altogether. With the limb properly aligned at the outset of lengthening, elective use of a telescoping intramedullary nail may now be favored over a frame accordingly.

## Background

Anisomelia is a clinical problem that is frequently referred to the pediatric orthopedist for management. The etiology is varied, ranging from congenital to acquired, and it may be characterized as static or progressive. The discrepancy may be localized in the femur, tibia, or both. The ilium and foot may also contribute to the overall measured discrepancy. Historically, scanograms were relied upon to provide accurate measurement. Computed tomography (CT) scan scout films are more accurate, but not universally available. The standing teleroentgenogram has emerged as the most popular assessment tool [1, 2]. This includes the pelvis and foot and allows for determination of the mechanical axis. More recently, the EOS imaging system provides simultaneous anteroposterior and lateral projection of the limbs, and may illustrate some of the rotational components.

The surgical armamentarium for managing anisomelia includes gradual limb lengthening versus acute shortening or epiphysiodesis of the longer limb. These may be utilized alone or in combination, depending upon the needs, tolerance, and resources of a given patient. The timing of intervention for equalizing limb lengths remains a subject of study and controversy. The classic guidelines for treating predicted discrepancy at maturity are listed in Table 1.

**Table 1:** Historical treatment guidelines

<1.5 cm	No intervention
1.5–5 cm	Epiphysiodesis
>5 cm	Limb lengthening (or shortening)

## Limb lengthening

Since the first femoral lengthening, performed by Codvilla in 1906, surgical lengthening of the foreshortened limb has been widely practiced. This is conceptually appealing and often favored by the parents because it preserves stature. Limb lengthening is typically accomplished by means of an external frame, secured to the bone segments with transfixion wires or half pins. As refined by Ilizarov, the typical rate of length gained is 1 mm per day. This method offers the advantages of gradually correcting not only length, but rotational and angular deformities. However, the pins, penetrating the skin and muscle compartments, may cause problems during the course of treatment. Adequate informed consent is difficult to achieve. Complications are common, sometimes serious, and often require unanticipated secondary procedures. Angular deformities may ensue due to bending of the regenerate bone upon removal of the frame and/or juxta-articular growth disturbance. If this method is employed during the growing years, recurrent discrepancy should be anticipated and may require secondary treatment. Once a child has had the experience of lengthening with a frame, they and their family will be reticent to repeat the process.

The comparatively recent development of telescoping intramedullary rods has solved the problems associated with transfixion pins. The most popular models are externally driven by electromagnetic control, with an average gain of 1 mm per day, often divided into three or four sessions. There are still potential complications, such as joint contracture or subluxation, premature consolidation, nonunion, etc., that require vigilance and management, in order to achieve the desired outcome. By necessity, lengthening occurs along the anatomic axis of the bone. Hence, the mechanical axis must be monitored and corrected, before, during, or after the lengthening.

## Epiphysiodesis

The option of inhibiting the longer extremity was introduced in 1933 by Phemister, who removed and rotated a rectangle of bone on each side of the physis, in order to produce a bone bridge [3, 4]. By definition, this is a permanent procedure and must, therefore, be well timed. With the advent of intraoperative fluoroscopy, this technique was refined to percutaneous drilling/curettage [5, 6]. Nevertheless, this latter method still demands perfect timing, because it is irreversible. Therefore, it is only appropriate for use in a narrow, adolescent age range.

In 1947, Blount proposed the revolutionary concept of instrumented, reversible epiphysiodesis with staples; this was applicable for both angular correction and length inhibition [7]. Problems inherent to stapling include migration, bending, or breakage of the implants, a reflection of the power inherent in physeal growth. During the ensuing decades, sophisticated technology and refined techniques, both simple and complex, have expanded our armamentarium for dealing with anisomelia.

In parallel with the evolution of hardware and techniques, improved methods have been developed for assessing skeletal maturity and estimating the optimal timing of surgical intervention. The Greulich and Pyle atlas and the Green–Anderson charts have been succeeded by such tools as the Moseley straight-line graph and, more recently, the Diméglio graph, and the multiplier method [8]. Historically, length inhibition has been recommended during the adolescent growth spurt, with hopes that a single operative intervention will suffice. When calculating “definitive” timing, modern algorithms represent important advances, but are still not without a margin of error [9–14]. Consequently, with permanent epiphysiodesis methods [Phemister, percutaneous drilling, and possibly percutaneous epiphysiodesis using transphyseal screws (PETS)], over- or under-correction of anisomelia may still occur. Furthermore, asymmetrical arrest may result in iatrogenic angular deformity that can only be resolved by osteotomy. The advent of tension band plating (2004), which is predictably reversible, has allowed us to intervene earlier than heretofore contemplated, because the physis can be untethered upon correction of the discrepancy [15–21]. This may be repeated, as necessary, depending upon the etiology of the condition and the ultimate discrepancy predicted. The concept of serial guided growth may be applied for both the ipsilateral angular deformities and to decelerate growth in the contralateral, longer limb.

## Applications for guided growth

**Fig. 1:**
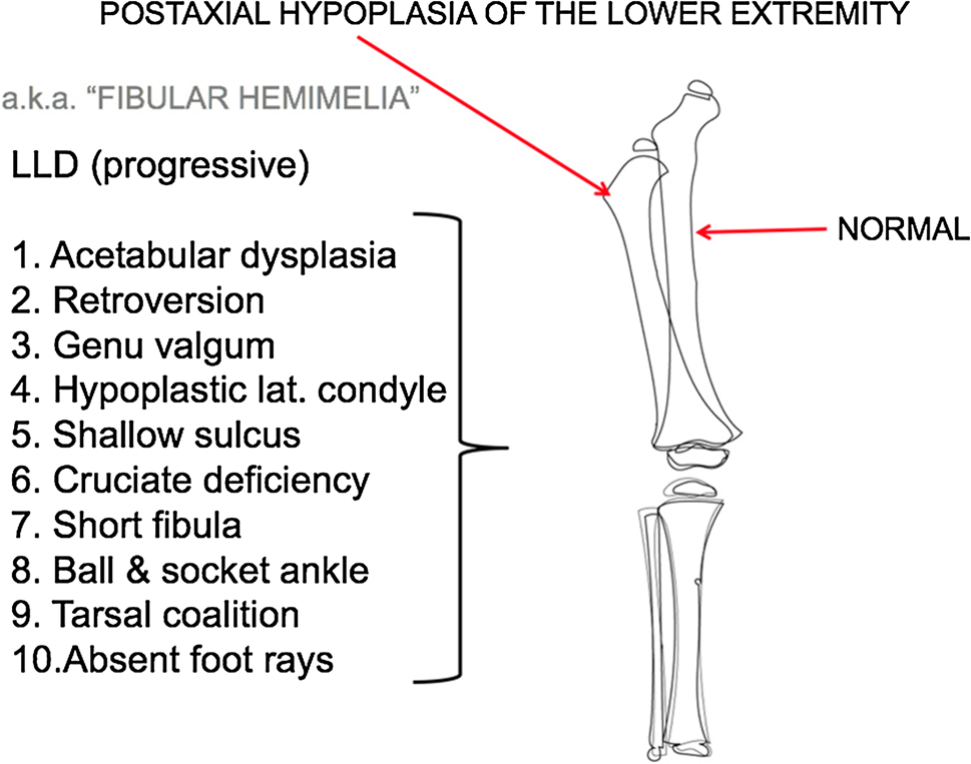
Postaxial hypoplasia may include any and all of the ten features listed. The oft used name “fibular hypoplasia” fails to implicate the components that contribute to this generalized limb dysplasia. These frequently complicate and compromise the outcome of limb lengthening

**Fig. 2:**
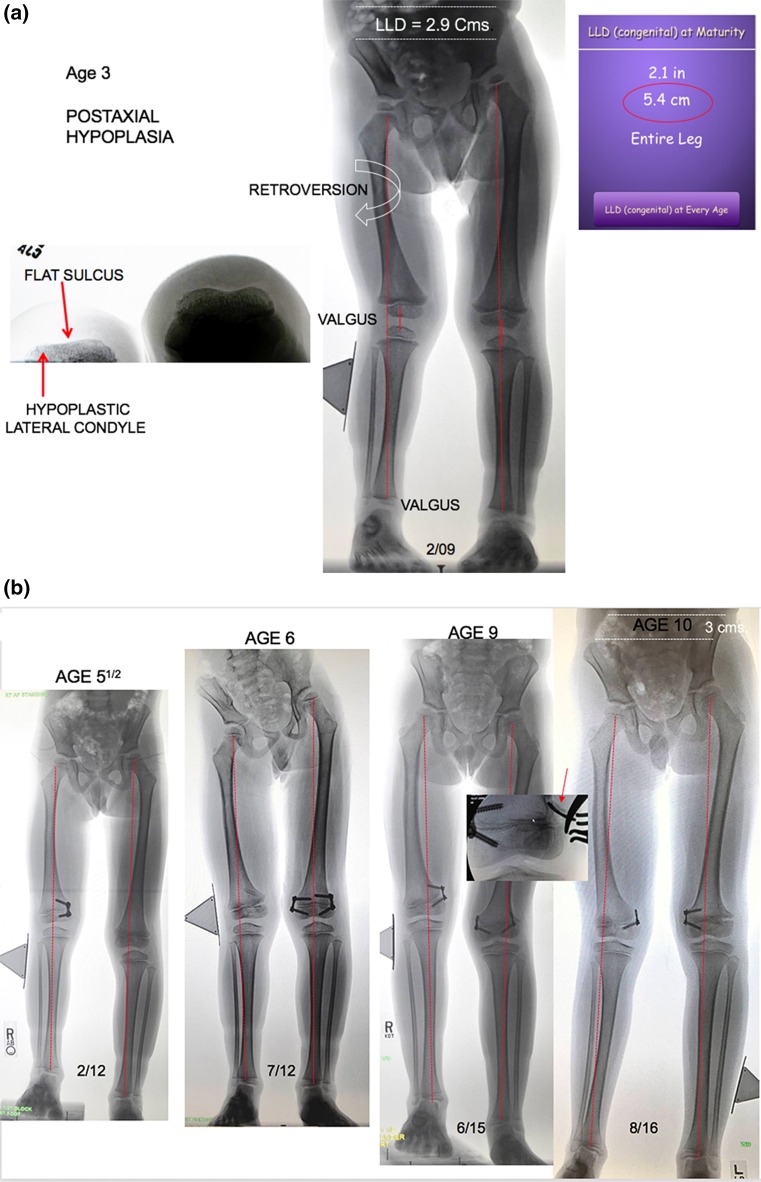

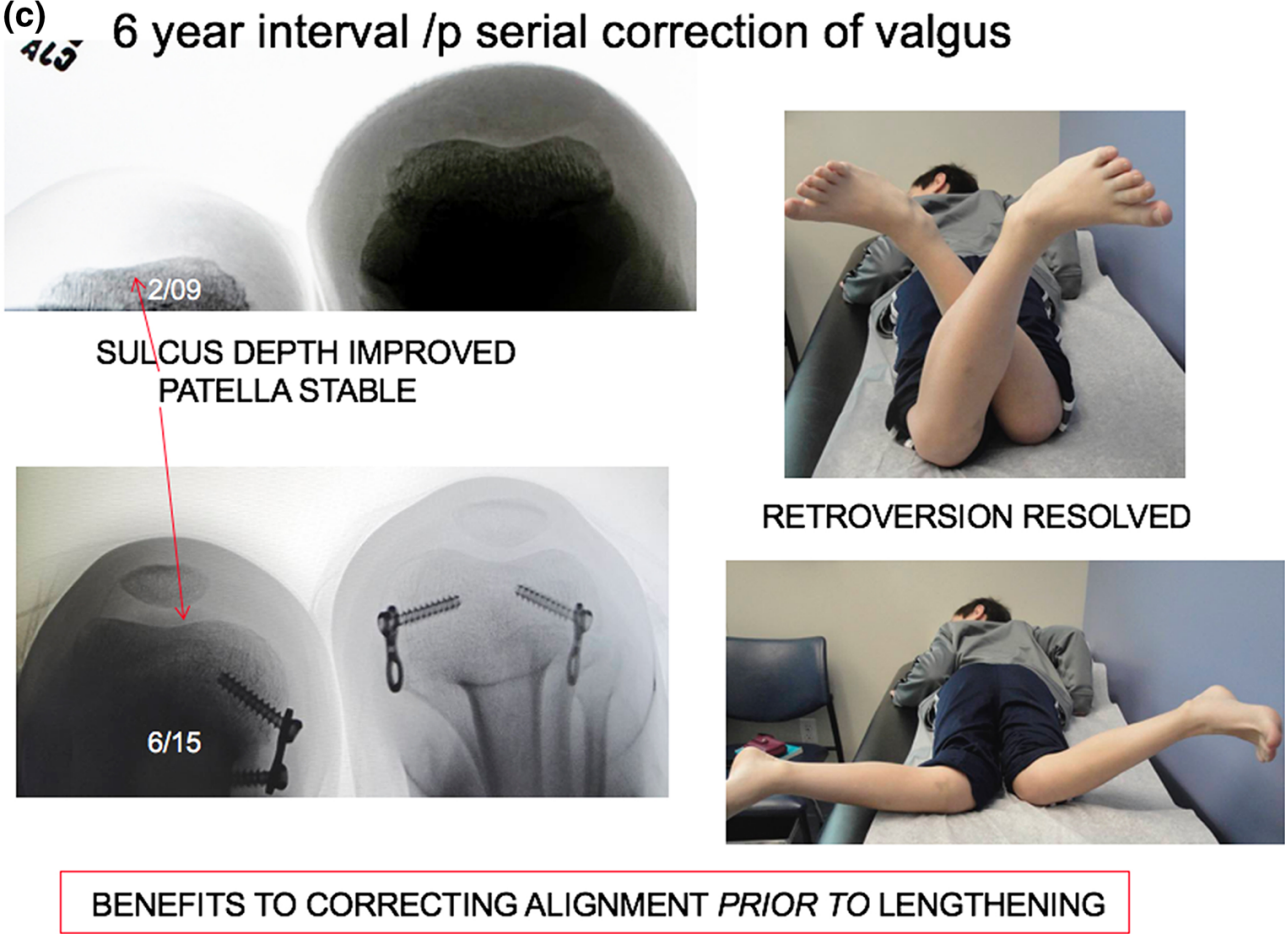
**a** This 3-year-old boy presented with a 2.9 cm limb length inequality and right knee pain. The cruciate ligament laxity, hypoplastic lateral femoral condyle, shallow sulcus, and retroversion together contribute to maltracking of the patella. **b** Commencing with guided growth of the medial right femur at age 3 years, this patient has undergone a series of outpatient surgeries with minimal scars and no functional limitations. Serial guided growth of the right distal medial femur has restored/maintained a neutral mechanical axis. Sequential tethering/untethering of the normal left femur has mitigated the limb length discrepancy. He will now undergo reinsertion of the metaphyseal screw on the right and addition of a lateral plate on the left. **c** In addition to the frontal plane angular adjustment, there are transverse plane benefits as well, including a deeper patella femoral sulcus and correction of femoral retroversion

The aforementioned guidelines for limb length equalization have been altered by recent developments. While it may seem counterintuitive to consider epiphysiodesis in the context of limb lengthening, there are specific situations where this combination makes sense:

1. *Gigantism* There are a number of syndromes that present with abnormal, accelerated growth of an extremity. These include Beckwith–Wiedemann syndrome, Klippel–Trenaunay or other hemangioma conditions, lymphedema, neurofibromatosis, epidermal nevus syndrome, Proteus syndrome, etc. It is illogical to procrastinate and observe annual, incremental overgrowth of the involved extremity, anticipating eventual lengthening of the normal extremity, in order to achieve limb length equality. This strategy would require skeletal maturity as a prerequisite in order to accurately measure the discrepancy that, in the interim, could reach 10 cm or more. A progressively taller shoe lift (or caliper) and annual visits are, at best, temporizing and unfulfilling for the parents, nor is it well tolerated to undertake an acute shortening of the involved femur or tibia/fibula. Problems such as wound healing, compartment syndrome, or sustained weakness may ensue, and continued growth will compromise the outcome.
2. *Postaxial hypoplasia* *(Fig.* 1 *)* This condition, otherwise known as fibular hemimelia, is subtle upon presentation at birth. Sometimes, the only obvious finding at birth is a missing fifth toe. However, during growth, many related issues become apparent. Among them is the development of a progressive anisomelia, reaching several centimeters at maturity. Additional problems, confined to the short leg, are acetabular dysplasia, femoral retroversion, progressive genu valgum, cruciate laxity (or absence), and patella-femoral maltracking or instability [22]. Each of these may contribute to knee subluxation during the course of femoral lengthening. Anticipation of the latter would suggest the use of distal medial femoral guided growth, repeated as necessary, during growth. By restoring and maintaining the mechanical axis to neutral, several of these potential problems are mitigated (Fig. 2). This strategy is also useful for dealing with the commonly associated ball and socket/ankle valgus that presents as pronation of the foot.

### Angular strategy

Progressive angular deformity will cause gait disturbance, including circumduction pattern (valgus) and waddling/Trendelenburg (varus). Secondary problems may emerge, including torsional deformity, ligamentous laxity, patellar instability, and exacerbation of limb length inequality. For these reasons, it is prudent to pursue early intervention and restore the mechanical axis.

There is no time limit for the tolerance of a flexible, extraperiosteal physeal tether. The timing of the initial intervention is determined not by age, but by mechanical axis deviation (lateral zone +2 or 3) on a full-length anteroposterior, weight-bearing radiograph, taken with the patella facing forward and a block under the short side to level the pelvis. The screws are placed relatively parallel, diverging over time. In more severe deformities, one may observe intentional reversed bending of the plate; it will not break however. The screws should be countersunk in order to be of low profile and lessen the (unlikely) chances of breakage. Upon recognition, a broken screw may be readily replaced. Patients should be seen at 3-month intervals, with comparison radiographs as indicated. Some relative length gain is realized as the leg becomes straight. Meanwhile, pain is alleviated, patellar tracking improves, and, surprisingly, retroversion may improve. When the mechanical axis is just past neutral (medial zone −1), the metaphyseal screw may be removed percutaneously. If a patient fails to return for timely follow-up and overcorrection is observed, reversal of the implant is a fallback option.

As growth continues, rebound valgus (both knee and ankle) is common in this condition; it is simply and efficiently managed by percutaneously reinserting the metaphyseal screw(s) [23]. Employing this strategy, it is safe to begin as young as 3 years of age and repeat guided growth as needed, following the child to maturity. If lengthening is ultimately warranted, it is helpful to have a properly aligned limb and stable knee from the outset. This may prevent the need for a frame or obviate a fixator-assisted osteotomy at the time of intramedullary rod insertion. If lengthening is undertaken prior to skeletal maturity and knee or ankle deformity ensue, guided growth may be employed to restore alignment, during or after the lengthening.

### Length strategy

What about the concept of inhibiting the longer leg to mitigate the ultimate discrepancy? In some cultures, and for some parents, tampering with the uninvolved leg is considered taboo. Parents object to the idea of “stunting their child’s growth”. That said, some families cannot afford the financial and emotional costs that are involved in limb lengthening. This is especially true when a frame is used and/or serial lengthening is required to achieve correction. Some families, when fully apprised of the aggregate costs and potential “obstacles/problems/complications” associated with lengthening, will opt to delay osteotomy and lengthening for as long as possible. They may prefer to forestall, in favor of a telescoping intramedullary rod during adolescence. In modest discrepancies, lengthening may be avoided altogether.

**Fig. 3:**
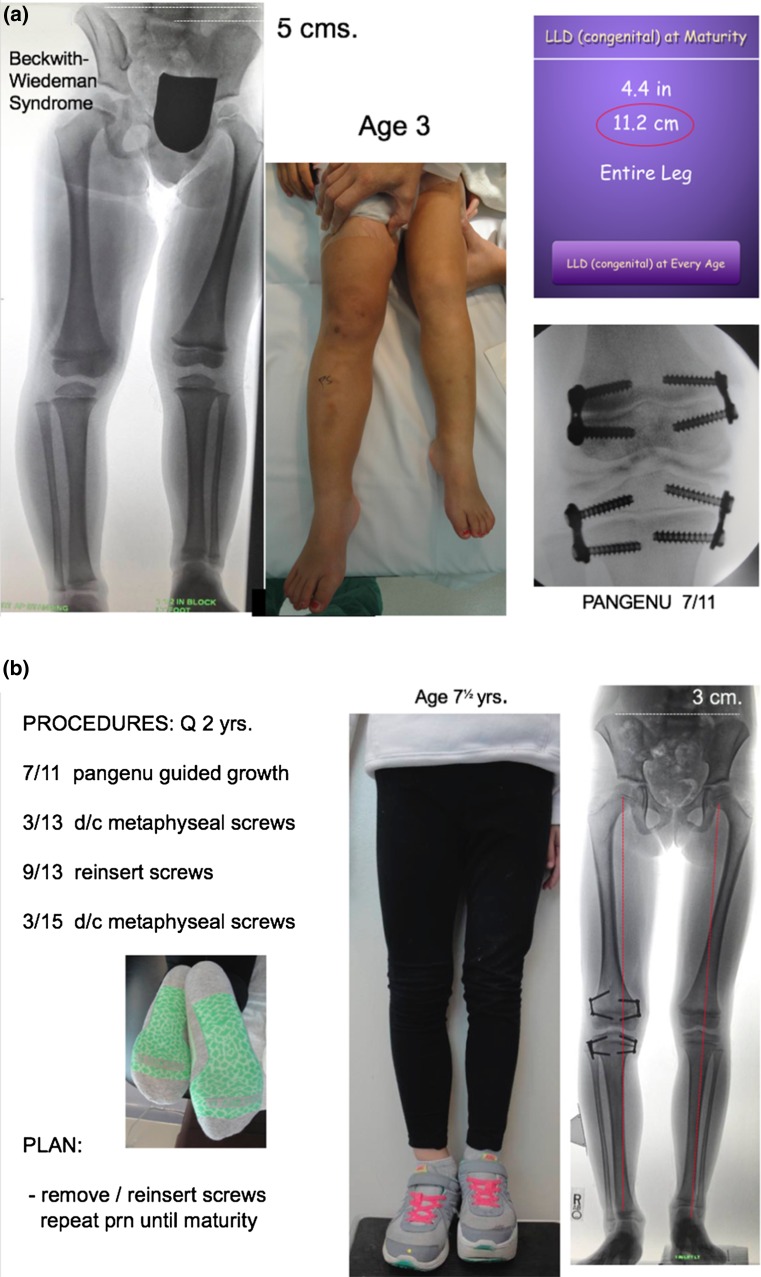

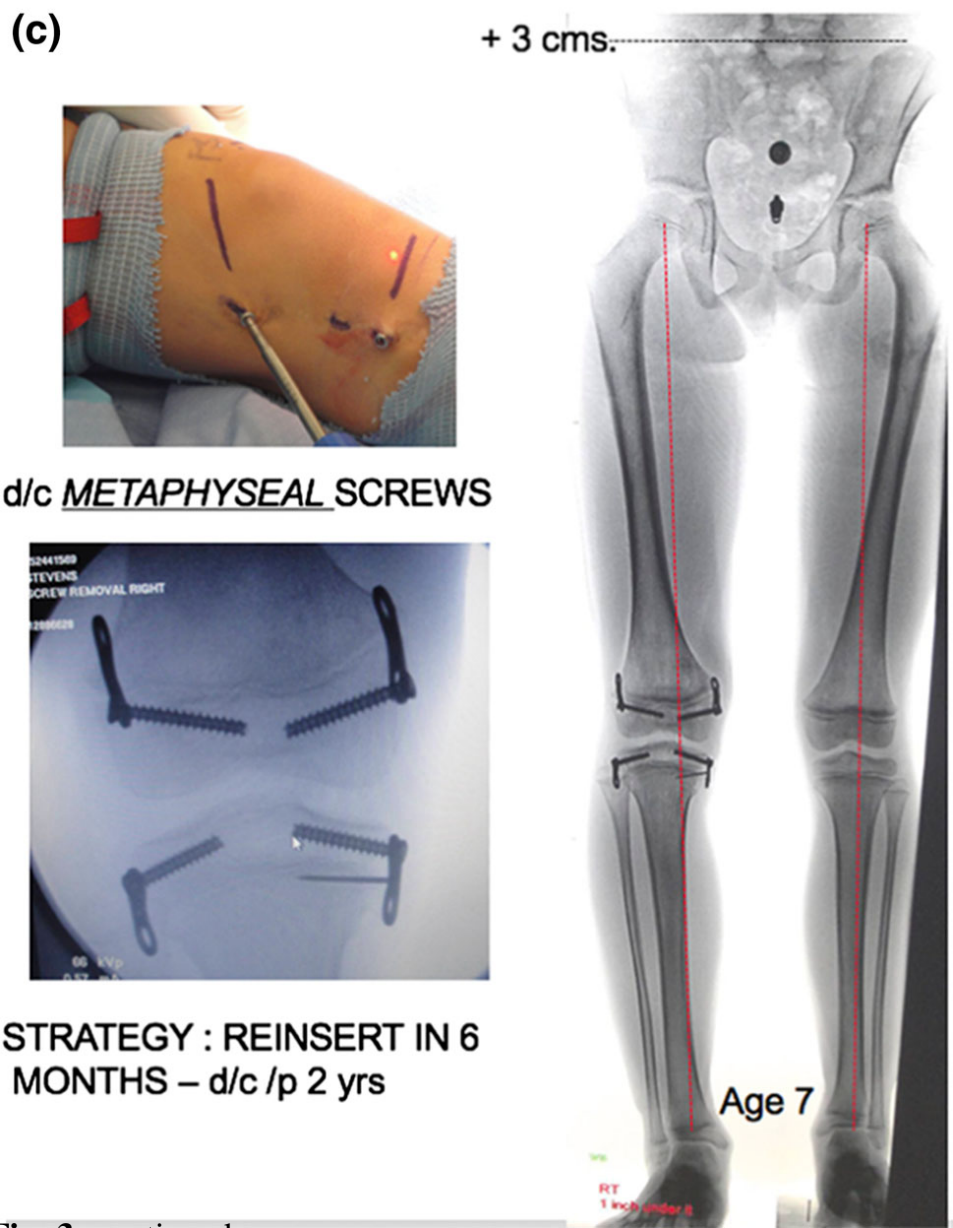
**a** This 3-year-old girl presented with a 5 cm discrepancy, estimated to progress to 11.2 cm at maturity. The strategy of serial growth deceleration was instituted. **b** Respecting the two-year tolerance for physeal restraint, the metaphyseal screws have been removed/reinserted and removed. She accommodates her discrepancy with a modest shoe lift. **c** Percutaneous screw removal. Depicted here is a trivial undertaking. At age 7 years, her discrepancy measures 3 cm, rather than the 7+ cm it would have been

When dual plates are applied to a physis to decelerate (not “arrest”) growth, there is an acknowledged two-year threshold, after which permanent physeal closure could occur. Therefore, the prudent strategy is to remove the metaphyseal screws by the two-year mark, wait six months to give the physis a reprieve, and reinsert them. Again, this process may commence as young as the age of 3 years and be repeated throughout growth. (Fig. 3) It is important to follow these children bi-annually, in order to detect any drift in the mechanical axis. When intercepted in a timely fashion, a screw may be removed and/or a plate reinserted. While the same plate/screw construct is utilized, it is advised to insert the screws in a moderately divergent fashion. If the screws are placed parallel to each other (as they are in angular applications), then the central physis may continue to grow, causing the screws to diverge. This implies a lag effect in the desired restraint of the physis. The divergent pattern, from the outset, circumvents this problem.

**Fig. 4:**
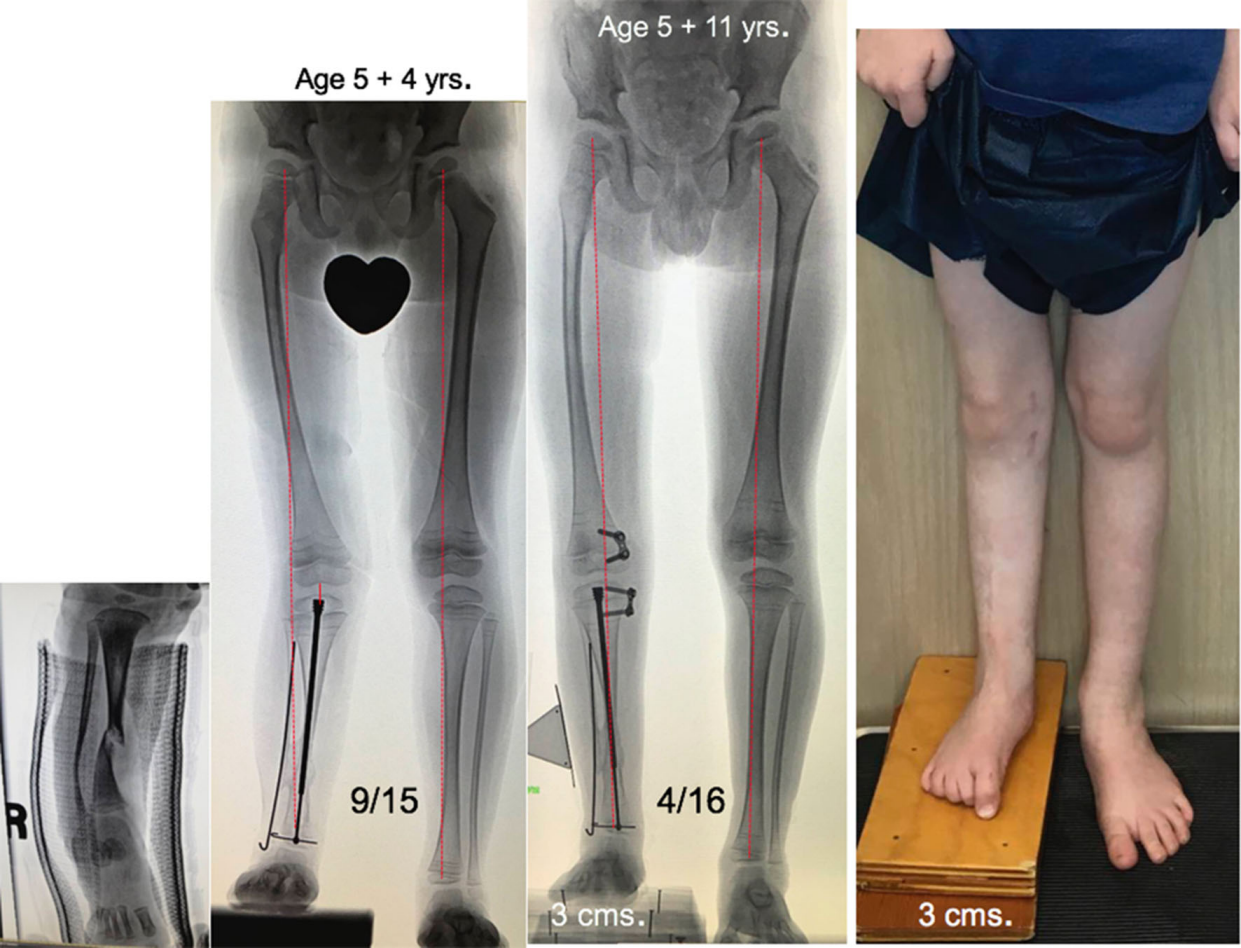
This neonate with NF1 was born with a tibial pseudarthrosis that required several surgical attempts at achieving union. By age 5 years, he presented with a united tibia, but progressive genu valgum, requiring hinged KAFO protection, and a 3 cm limb length discrepancy. Seven months following pan genu guided growth, the mechanical axis has been restored and bracing facilitated. The metaphyseal screws were removed and will likely be reinserted in the future. Per parental discretion, tethering of his left tibia is an option, in addition to the planned, eventual lengthening of his right tibia/fibula

3. *Adjunct to lengthening* *(Fig.* 4 *)* Before or concurrent with limb lengthening, correction of knee and/or ankle alignment may be achieved by guided growth, thus simplifying the construct of a given frame. During lengthening, or subsequent to frame removal, it is not uncommon for varus (femur) or valgus (tibia) to develop and compromise the outcome. Despite prophylactic bracing, deformity may present, due to bending or fracture of the regenerate bone, or it may occur through the physes. If recognized promptly, the situation may be salvaged by means of guided growth, rather than having to reapply the frame. In the sagittal plane, fixed knee flexion deformity or ankle equinus may be addressed by means of anterior distal femoral plates or a distal tibial plate, respectively.
4. *Limb salvage* Pediatric oncologic surgeons sometimes must perform major limb-sparing operations that may sacrifice one or more physes and/or the entire knee joint. As the child continues to grow, progressive limb length discrepancy poses functional and gait problems that are progressive. Although expanding prostheses have improved, they still have their limitations. This situation may be mitigated by pan genu epiphysiodesis of the uninvolved extremity.

## Conclusion

Considering technological advances, both in limb lengthening and growth inhibition, the classic guidelines, indications, and timing for the use of each method are being redefined. The versatility of frame lengthening, be it unilateral, hybrid, or hexapod, has greatly increased their usage. However, related complications and rising cost remain a challenge. Recent and improved iterations of telescoping intramedullary rods have challenged the predominance of frames.

Meanwhile, the advent of reversible guided growth technology nicely compliments the above lengthening techniques. Alignment can be restored before, during, or after lengthening. Lengthening may be postponed, reduced in frequency, or, in some cases, averted altogether by means of intermittent guided growth. It behooves the surgeon to be familiar with both modalities. Education of the parents and routine follow-up until maturity are paramount to success. Involving the parents in informed decision-making will empower them to choose the best combination and timing of procedures to address their child’s problems, while minimizing aggregate cost and potential complications.
